# Costs of two vancomycin-resistant enterococci outbreaks in an academic hospital

**DOI:** 10.1017/ash.2022.365

**Published:** 2023-01-13

**Authors:** Simon van der Pol, Mariëtte Lokate, Maarten J. Postma, Alex W. Friedrich

**Affiliations:** 1 Department of Health Sciences, University Medical Center Groningen, Groningen, The Netherlands; 2 Health-Ecore, Zeist, The Netherlands; 3 Department of Medical Microbiology and Infection Control, University Medical Center Groningen, Groningen, The Netherlands; 4 Department of Economics, Econometrics and Finance, University of Groningen, Groningen, The Netherlands; 5 Institute of European Prevention Networks in Infection Control, University Hospital Münster, Münster, Germany

## Abstract

**Objective:**

In early 2017, the University Medical Center Groningen, the Netherlands, had an outbreak of 2 strains of vancomycin-resistant enterococci (VRE) that spread to various wards. In the summer of 2018, the hospital was again hit by a VRE outbreak, which was detected and controlled early. However, during both outbreaks, fewer patients were admitted to the hospital and various costs were incurred. We quantified the costs of the 2017 and 2018 VRE outbreaks.

**Design:**

Using data from various sources in the hospital and interviews, we identified and quantified the costs of the 2 outbreaks, resulting from tests, closed beds (opportunity costs), cleaning, additional personnel, and patient isolation.

**Setting:**

The University Medical Center Groningen, an academic hospital in the Netherlands.

**Results:**

The total costs associated with the 2017 outbreak were estimated to be €335,278 (US $356,826); the total costs associated with the 2018 outbreak were estimated at €149,025 (US $158,602).

**Conclusions:**

The main drivers of the costs were the opportunity costs due to the reduction in admitted patients, testing costs, and cleaning costs. Although the second outbreak was considerably shorter, the costs per day were similar to those of the first outbreak. Major investments are associated with the VRE control measures, and an outbreak of VRE can lead to considerable costs for a hospital. Aggressively screening and isolating patients who may be involved in an outbreak of VRE may reduce the overall costs and improve the continuity of care within the hospital.

Enterococci are bacteria normally present in the human gastrointestinal system. Especially in healthcare settings, enterococci resistant to certain antibiotics are transmitted, most importantly vancomycin-resistant enterococci (VRE).^1^ The number of resistant isolates varies by country. In continental Europe, low resistance generally is found in northern and western countries, while high resistance in found in the east and south.^2^ In the Netherlands, the prevalence of VRE is low compared to most European countries, ranging from 0% to 2% of clinical isolates.^2^ VRE is mainly transmitted through contaminated surfaces and the hands of healthcare workers^3,4^; hence, VRE transmission can be reduced by strictly isolating patients carrying the resistant bacterium and by adhering to hygiene guidelines, such as frequent handwashing.^5^ An important tool to adequately isolate VRE-carrying patients concerns the screening of high-risk patients, such as patients suffering from gastrointestinal diseases or patients who have been admitted to hospitals in regions with a high prevalence of VRE. Most patients carrying VRE will not become ill due to the resistant bacterium; however, when the VRE causes an infection, it may more difficult to treat compared to susceptible enterococci and result in worse health outcomes, especially for patients with comorbid conditions.^1,6^ A recent study estimated the global number of deaths caused by various enterococci to be almost 100,000 in 2019.^7^

Early in 2017, the University Medical Center Groningen (UMCG) in the Netherlands had an outbreak of VRE, defined as transmission of a specific subtype of VRE to several patients in a defined period and location. Due to an incidental finding during routine screening from a patient who was hospitalized for ∼10 days, patients who shared the same hospital room were screened. Prior to the outbreak, patients previously admitted to hospitals abroad or patients admitted to the gastrointestinal ward were screened. In the months leading up to the outbreak, on average, 9 patients per day were screened, with a positivity rate of 0.6%. During the outbreak, 68 tests were performed per day and 2.8% were positive for VRE. Typing with next-generation sequencing (NGS) showed that 2 strains were causing the outbreak. One of these strains was isolated first from a patient who had previously been admitted to a German hospital and tested positive in November 2016. Despite isolation measures, the VRE strain could spread during December 2016 and the first weeks of January 2017. Due to the movements of patient to various wards and intensive care units, VRE spread to several locations.

At UMCG, 2 wards with many positive patients were closed completely and the patients had to be moved to a temporary ward that was only used for VRE-positive or high-risk patients (ie, patients who tested negative at the initial sampling but shared rooms or facilities with positive patients). In UMCG, most rooms are shared rooms. To prevent further transmission and to ensure adequate capacity in the intensive care for acute care, new admissions to the hospital were stopped. In total, 38 patients tested positive for VRE during this outbreak, with 2 separate strains. During the summer of 2018, 27 patients tested positive in another outbreak, all of whom could be traced back to a single VRE-carrying patient. Again, despite isolation measures, the VRE spread. To contain the spread of VRE, new patients were temporarily rejected, current patients were moved to an outbreak ward and the original ward, was completely disinfected using hydrogen peroxide vapor decontamination. Because the outbreak was detected in an early stage and aggressive measures were taken to clean the affected ward, the outbreak was controlled quickly without spreading to other wards within the hospital. During the first outbreak, no invasive VRE infections occurred in the patients who tested positive for VRE. During the second outbreak, the index patient had a positive blood culture, indicating an invasive VRE infection.

Few studies previously assessed the costs associated with VRE outbreaks in hospitals. A VRE outbreak in the UMCG in 2013 was estimated to cost ∼€3800 (US $4,034) per day^8^ and in a study in a French university hospital the total costs of a VRE outbreak amongst 13 patients was estimated at €171,439 (2008 euros; US $182,457), with the opportunity costs being a major driver.^9^

During these outbreaks, various costs were incurred by the hospital, such as the costs of cleaning, personnel costs, laboratory costs and lost costs due to closed beds. In this research, we quantified the costs directly attributable to the 2017 and 2018 VRE outbreaks at UMCG.

## Methods

The UMCG is an academic hospital in the Netherlands with >28,000 admissions annually, a workforce of >11,000 full-time equivalents, and an annual revenue of €1.6 billion (US $1.7 billion).^10^ In a prior study, in which costs associated with several outbreaks in the UMCG were quantified, 5 main categories of costs were identified: diagnostics, closed beds, cleaning, additional personnel, and patient isolation.^8^ These categories were also assessed in this study. To calculate the costs associated with each category, we estimated volumes of the various categories and multiplied them with the unit costs. To estimate the volumes of the various items, we used clinical data and data collected during interviews with representatives of various departments that were affected by the outbreaks.

The first outbreak started January 10, 2017, and ended February 21, 2017. The second outbreak started August 21, 2018, and ended September 8, 2018. For both outbreaks, transmission occurred before the starting date. We considered only the periods when hospital staff were aware of the outbreak.

The methods for sampling and culturing VRE in the UMCG are described in detail elsewhere.^11^ For the analyses, the VRE status of patients is important, and we used 3 categories: (1) VRE positive, for patients confirmed to carry VRE, having positive results in both a polymerase chain reaction (PCR) test and a culture; (2) VRE suspect, for patients at high risk to carry VRE, including patients sharing rooms with VRE-positive patients, and in case VRE was spread across several rooms, everyone admitted to an affected ward for >24 hours; and (3) VRE negative, for patients not suspected of carrying VRE.

### Data sources

*Interviews.* Overall, 8 interviews with representatives of relevant departments were conducted to get an overview of the relevant costs. We interviewed staff from the infection prevention unit, the microbiological and viral laboratories, the most severely affected department (ie, the gastrointestinal unit), facility services, procurement, and business intelligence. These interviews were used to get an expert opinion on various costs, but interviewees were also requested to provide data sources where available.

*Data sources.* Various sources of clinical input data were used to assess the unit volumes used for the cost analysis. Additional hours worked by nurses were registered by the planning department. Patient movement data were used to estimate the number of times a room had to be cleaned. Cleaning staff was estimated to need 1–2 hours to complete cleaning per room in which a VRE-positive patient was admitted. The VRE decontamination procedure for these rooms included spraying the room with a chlorine solution, disinfecting the beds and medical devices, removing unwrapped disposables, and replacing the curtains. Some patients were admitted to rooms where >1 VRE-positive patient was admitted; therefore, we assumed that the cleaning time per VRE-positive patient was 1 hour. Patient isolation data were used to estimate the number of days VRE-positive or VRE-suspect patients were put in isolation to prevent further spread through the hospital. Ward occupancy data were used to estimate the opportunity costs due to closed beds.^12^ Laboratory data were used to estimate the volume of VRE tests performed, from both patient and environmental samples.

To estimate the unit costs, Dutch reference prices^13^ were used where possible; otherwise, data from previously published literature were used. Table 1 provides an overview of the various unit costs, including references. All costs were converted to 2020 euros using the health-related consumer price index, as published by statistics Netherlands.^14^ Internal cost calculations from the microbiology department were used for the unit prices of the various tests. These are the costs that are offset to the clinical departments; they include staff costs but do not reflect commercial prices as all tests were performed by the internal laboratory of the UMCG. Cleaning costs per hour were used as published in previous research.^8^ For the 2018 outbreak, the affected ward was cleaned using hydrogen peroxide; the invoice totals were used in the analysis.

**Table 1.** Unit Prices, Expressed in 2020 Euros

Item	Price, Euros	Price, US $^a^	Reference
Ward stay (academic hospital), per day	662.87	703.88	Hakkaart-van Roijen et al^13^
Intensive care unit stay, per day	1,224.55	1300.31	Hakkaart-van Roijen et al^13^
Contact isolation, per patient per day	26.27	27.90	Dik et al^8^
Cleaning costs (weekday or weekend day), per hour	26.52	28.16	Dik et al^8^
Hourly labor costs: infection and prevention specialist	39.28	41.71	Dutch federation of university medical centres^15^
Hourly labor costs: nurse during regular hours	36.36	38.61	Dutch federation of university medical centres^15^
Hourly labor costs: nurse during night shifts and on Saturday	53.45	56.76	Dutch federation of university medical centres^15^

a Currency conversion as of December 28, 2022.

### Opportunity costs due to closed beds

During both outbreaks, patient admissions in the UMCG were stopped, making it likely that revenue was missed. To estimate the missed revenue for both outbreaks, we used occupancy rates of the patient wards in the hospital, and we compared the real occupancy with the expected occupancy from the start of the outbreak until 2 weeks after the outbreak to account for a lag period after reopening the wards. This expected occupancy was estimated using autoregressive integrated moving average (ARIMA) models.^16^ For each ward, the best-fitting ARIMA model was automatically determined using the Hyndman-Khandakar algorithm.^17^ To estimate the opportunity costs, we used the difference between the measured occupancy and the bootstrapped 95% prediction interval of the forecast occupancy.^16^ For example, if the lower bound of the ARIMA model predicted an occupancy of 10, but only 6 patients were admitted to a ward, the difference (10 – 6 = 4) was used to calculate the opportunity costs due to closed beds. The result was then multiplied by the ward stay costs to estimate the opportunity costs (Table 1). No nursing personnel costs were subtracted from the costs because nurses from closed departments were relocated to different departments. Also, personnel deployment was less efficient as patients were spread through the hospital, and there was an overall shortage of personnel. The various fit models, including the prediction intervals and the measured values, are displayed in Supplementary Figures 1 and 2.

During both outbreaks, a transfer ward was established to isolate VRE-positive patients. To prevent the double counting of outbreak-related costs, we did not include the hospitalization costs of this transfer department in the analysis because they are counted using the ARIMA models.

### Cleaning and patient isolation costs

For all VRE-positive patients, we assumed 1 hour of cleaning for every movement through the hospital, from the moment at which the VRE infection was confirmed. For all VRE-suspect patients, we also assumed 1 hour of cleaning for every movement through the hospital, until they were confirmed to be VRE negative. If this exact point of time was unknown in the patient isolation records, we assumed a period of 48 hours between the start of the suspicion of VRE and the confirmation of the VRE status, either positive or negative. Patient isolation costs were applied to all patients, for each isolation day.^8^

### Test costs

Test costs were calculated by multiplying the number of tests performed with the costs per test. After the 2017 outbreak, an inhouse PCR test was developed, resulting in a less expensive test used in the 2018 outbreak, compared to the 2017 outbreak, where the Cepheid GeneXpert (Cepheid, Sunnyvale, CA) was used. A patient’s first positive PCR result was followed by NGS. VRE tests are performed regularly in the UMCG. To correct for the baseline level of VRE tests, we used the average daily number of VRE tests from the preceding 4 months and subtracted this number from the total tests during the outbreaks. For example, if the average daily tests in the 4 months prior to the first outbreak was 9 and the average number of tests per day during the outbreak was 68, then a total of 68 − 9 = 59 daily tests were counted toward the outbreak costs. Due to the confidentiality of the unit prices for tests, only aggregated costs were reported.

### Analyses

All analyses were performed using R version 4.1.0 software (R Foundation for Statistical Computing, Vienna, Austria) with the dplyr package for data transformation.^18,19^ For time series analyses, the fable package was used.^20^ In addition to the total costs of both outbreaks, the outbreak costs per day were calculated.

### Ethical statement

The Medical Ethical Committee of the University Medical Center Groningen (UMCG) exempted the present study from a full review according to the Dutch Medical Research with Human Subjects Law because it falls outside the scope of this law. This research was approved by the UMCG Central Ethics Review Board and was considered to adhere to all applicable laws, such as the Medical Treatment Agreement Act, Data Protection Act and the Code of Conduct for Responsible Use.

## Results

**Fig. 1.**
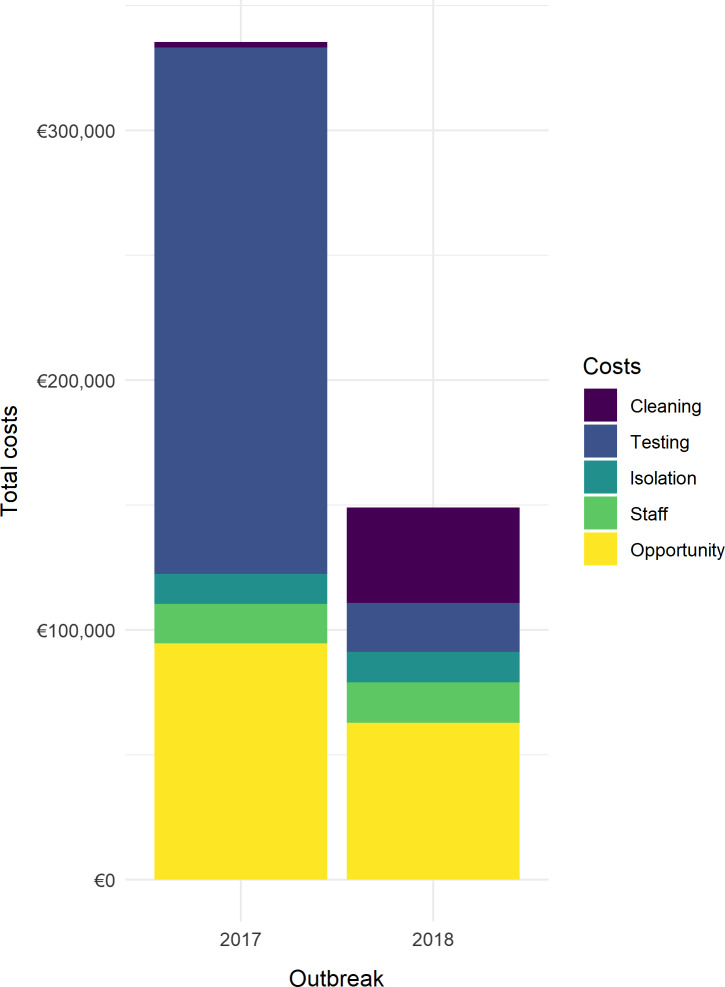
Schematic overview of costs related to VRE outbreaks in 2017 and 2018.

During the first outbreak, in 2017, 38 patients tested positive for VRE. In the second outbreak in 2018, 27 patients tested positive for VRE. Table 2 provides an overview of the resources used during both outbreaks. Table 3 summarizes the costs associated with the 2017 and 2018 outbreaks, which are graphically displayed in Figure 1. The total costs associated with the 2017 outbreak are estimated at €335,278 (US $356,826) or €7,983 (US $8,496) per day. The total costs associated with the 2018 outbreak are estimated at €149,025 (US $158,602) or €7,843 (US $8,347) per day. The main driver of the costs in the 2017 outbreak are the diagnostics, followed by the opportunity costs due to closed beds. For the 2018 outbreak, the opportunity costs are the main driver, followed by the cleaning costs. A large proportion of the costs in 2018 concerns the hydrogen peroxide cleaning costs, which amounted to >25% of the total costs during this outbreak.

**Table 2.** Overview of Increased Resource Use During the 2017 and 2018 Outbreaks

Variable	First Outbreak	Second Outbreak
Patients tested positive for VRE	38	27
Additional hours worked by nurses	202	292
Rooms cleaned^a^	97	71
Isolation days	456	465^b^
Additional PCR tests performed	3673	324
Cultures performed	117	52
NGS tests performed	38	27

Note. VRE, vancomycin-resistant enterococci; NGS, next-generation sequencing; PCR, polymerase chain reaction.

a Excluding hydrogen peroxide cleaning in the 2018 outbreak.

b Includes 1 outlier with a length of stay of >5 months.

**Table 3.** Costs Associated with 2017 and 2018 VRE Outbreaks, Total Costs, and Percentage of Total Outbreak Costs^a^

Variable	2017			2018		
	Euros	US $^b^	%	Euros	US $^b^	%
Cleaning						
Cleaning staff	2,175	2,309	0.7	1,671	1,774	1.1
Hydrogen peroxide	NA	NA		36,551	38,812	24.5
Testing	210,733	223,771	62.9	19,569	20,780	13.1
Isolation	11,981	12,722	3.6	12,217	12,973	8.2
Staff	15,789	16,765	4.7	16,260	17,266	10.9
Opportunity costs due to closed beds	92,601	98,330	28.2	65,756	69,824	42.1
Total	335,278	35,6021	100	149,025	158,245	100
Total per day	7,983	8,477		7,843	8,328	

a All costs are rounded to full euros, percentages are rounded to 1 decimal place.

b Currency conversion as of December 28, 2022.

## Discussion

In this study, we assessed the outbreak costs of 2 VRE outbreaks in the UMCG (Groningen, The Netherlands). The total costs of the 2017 outbreak were estimated at €335,278 (US $355,914) and the total costs of the 2018 outbreak were estimated at €149,025 (US $158,197). The main drivers of the costs were the opportunity costs, additional diagnostics, and cleaning costs. Although the second outbreak was considerably shorter, the costs per day were similar to those of the first outbreak. Compared to the first outbreak, cleaning costs were considerably higher during the second outbreak. However, due to the early intervention and isolation measures, fewer wards needed to be screened for VRE, resulting in a major decrease in PCR tests (by a factor of 10). Although the first outbreak was longer, fewer additional hours were worked by nurses. This difference may have been due to a constrained availability of staff because no additional nurses could be found. The overall reduction in costs for the 2018 outbreak may be due to the early, intensive response, which may have prevented a hospital-wide outbreak that could have resulted in overall costs comparable to the 2017 outbreak.

Compared to a previous study of a VRE outbreak in the UMCG, the costs per day were considerably higher in this study: ∼€7,900 (US $8,407) compared to €3,800 (US $4,044) in 2013, the latter amounting to €4,100 (US $4,363) in 2020 euros.^8^ However, this outbreak was smaller; 19 patients were involved and only 1 ward was affected. Like the 2017 and 2018 outbreaks, the major drivers of these costs were those of diagnostics and opportunity costs due to closed beds.^8^ The French study also estimated that the loss of income from spare isolation beds was a major driver of the overall outbreak costs.^9^ A difference in this study with ours concerns the inclusion of the costs of antibiotics, which was the second most important driver of costs. We also considered the inclusion of antibiotics; however, the main alternative for VRE patients is teicoplanin, and the price differences between vancomycin and teicoplanin in the Netherlands are negligible. Thus, we decided to not include these as extra costs.^21^

This study had several limitations. It is complex to accurately estimate the opportunity costs due to closed beds because patients are exchanged between the various wards in the hospital. Also, we were unable to determine exactly how many patients went to other hospitals during the period of no new admissions at UMCG. We tried to estimate this cost using various ARIMA models, and we considered the measured occupancy outside the 95% prediction intervals to be caused by the outbreaks. This is a conservative approach; the prediction intervals are rather wide due to the variability in the data. Hence, we may have underestimated the opportunity costs due to closed beds. In December 2017, the UMCG switched the computer system used to measure the ward occupancy and movements of patients through the hospital, resulting in poorly comparable data for the 2 periods. We trained the time-series model on the 4 months preceding both outbreaks to make sure the data cut caused by the new system did not affect the analysis, but this prevented us from fitting more advanced predictive models.

Another limitation related to the cleaning costs. Although the cleaning procedures for patients in contact isolation are rather strict, no data were available regarding increased staff expenditure and cleaning materials. Instead, an approximation was used in which we counted 1 hour of cleaning time per isolated patient. The cleaning costs in the 2018 outbreak were higher compared to the 2017 outbreak. In the 2018 outbreak, hydrogen peroxide vapor decontamination was used to ensure a rapid reopening of the ward. This was very precisely accounted for in this analysis because the invoice was available. Although the costs associated with hydrogen peroxide vapor decontamination are substantial, we did not assess its effectiveness relative to standard cleaning procedures. The overall costs of the second outbreak may have been lower if cleaning staff had thoroughly cleaned the affected ward instead of the specific approach with hydrogen peroxide. The limitations concerning data collection raise an important opportunity to improve the registration and availability of data. Even for directly involved staff, high-quality data were difficult and in many cases impossible to find.

We did not consider clinical consequences of VRE-positive patients because we focused on the direct costs attributable to the outbreak. A meta-analysis including 12 cohort studies found increased mortality for patients with a VRE bacteremia and increased length of stay compared to vancomycin-susceptible enterococci (VSE).^6^ Currently, the incidence of VRE is low in the Netherlands,^2^ and the stringent VRE control measures may have played a role. Our results indicate that major investments are associated with VRE control measures. However, the containment of VRE may result in lower healthcare costs overall due to a shorter length of stay and decreased mortality. Estimations from previous research show that annually ∼16,000 VRE infections occur in Europe, causing >1,000 deaths, but the burden of VRE-related morbidity and mortality in the Netherlands is very low.^22^

From this study, we can conclude that an outbreak of VRE can lead to considerable costs for a hospital. Although each outbreak differs, aggressively screening and isolating patients who may be involved in an outbreak of VRE may reduce the overall costs and improve the continuity of care within the hospital.

## Supplementary material

For supplementary material accompanying this paper visit https://doi.org/10.1017/ash.2022.365. click here to view supplementary material
