# Supplementary material for: Costs of two vancomycin-resistant enterococci outbreaks in an academic hospital
Source: Antimicrob Steward Healthc Epidemiol. 2023 Jan 13;3(1):e8. doi: 10.1017/ash.2022.365 (PMC9879878; doi:10.1017/ash.2022.365)

Supplementary figure 1: Time series used for opportunity costs 2017 outbreak

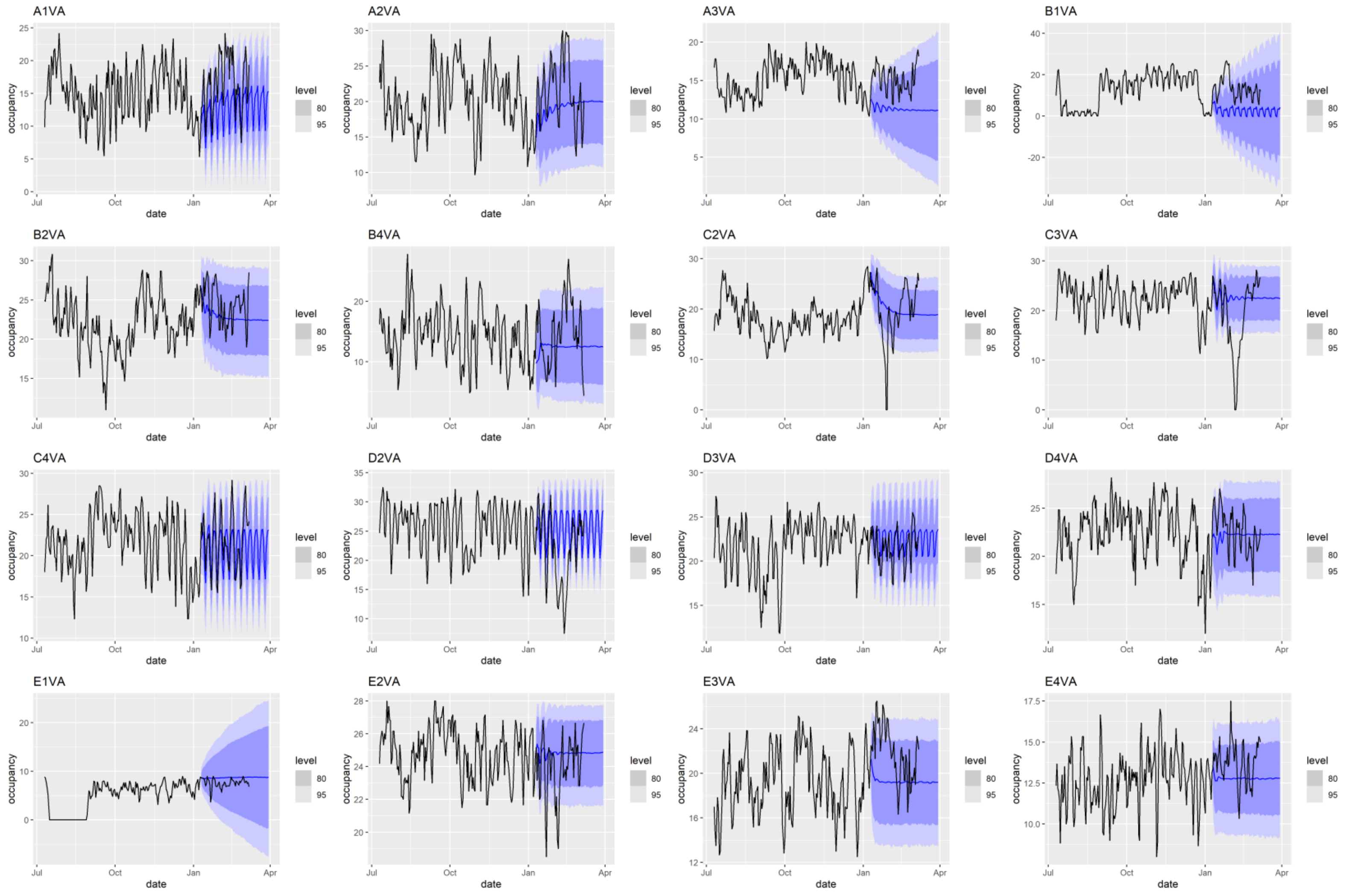

Supplementary figure 2: Time series used for opportunity costs 2018 outbreak

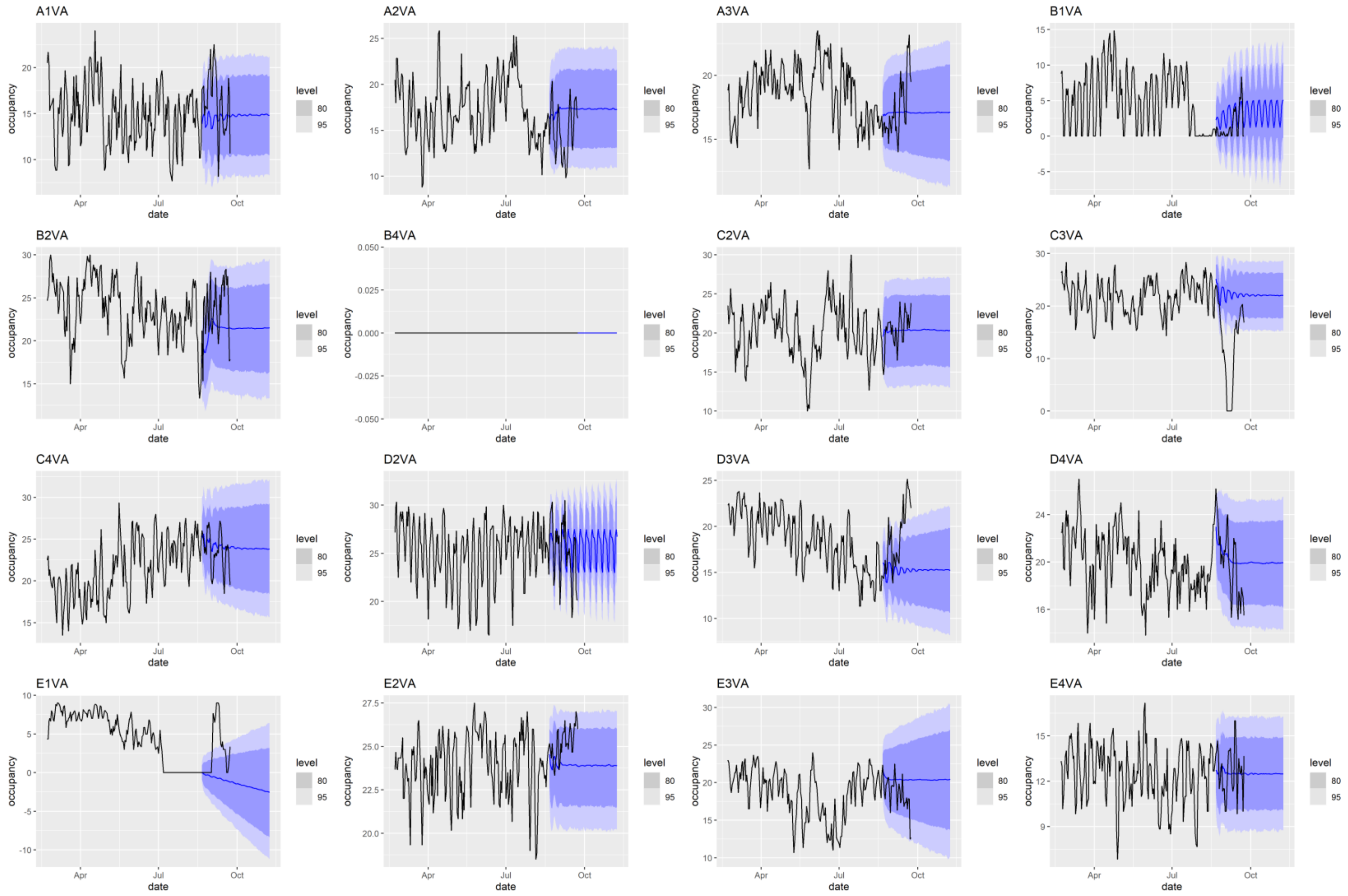

Supplement: Supplementary file 1 [file S2732494X22003655sup001.pdf]
